# Real-word evidence on the effectiveness of the Endo-App in reducing endometriosis-related complaints: a retrospective analysis

**DOI:** 10.1007/s00404-026-08494-0

**Published:** 2026-07-27

**Authors:** Meletios P. Nigdelis, Christine Anderl, Erich-Franz Solomayer, Martin Sillem

**Affiliations:** 1 Department of Gynecology, Obstetrics, and Reproductive Medicine, Saarland University Medical Center, Homburg, Germany; 2 Endo Health GmbH, Chemnitz, Germany

**Keywords:** Endometriosis, Digital health, App, Real-world evidence, Pelvic pain

## Abstract

**Purpose:**

Randomized controlled clinical trials have shown that symptoms of chronic pain conditions, such as endometriosis, can be improved with the use of specialized health apps. In the current study, real-world evidence was collected from Endo-App users to determine whether there was a sustained and positive effect on endometriosis symptoms.

**Methods:**

Users of the Endo-App were offered an optional, six-question anonymous survey through the platform upon first registration (T0), after 3 (T1), and 6 to 7 (T3) months of use. The survey questions were not standardized and aimed to assess the severity of endometriosis symptoms and overall quality of life. Data were analyzed using MatLab using a parametric paired t-test when pairs of data between T0–T1 and/or T0–T2 were available. Statistical significance was defined as *p* < 0.05.

**Results:**

A total of 143 users of the app completed at least part of the survey at 3 months and 55 users at 6 to 7 months. After 3 and 6 to 7 months of using the app average pain levels and days requiring medical assistance were significantly reduced compared to baseline values. In addition, the average reported number of sick days taken was significantly reduced at the 6-to-7-month timepoint compared to baseline.

**Conclusion:**

This real-world study demonstrates that use of a specialized app for endometriosis, such as the Endo-App, documents and may lead to a significant and sustained improvement of some endometriosis symptoms.

## What does this study add to the clinical work?

**Table:** 

This study demonstrates that the Endo-App might be associated with lasting symptomatic improvement in patients diagnosed with endometriosis. These results are based on real-world evidence and should be investigated across larger populations.	

## Introduction

Endometriosis is a chronic inflammatory condition that is identified by the presence of endometrial-like tissue outside the uterus [1–4]. It affects ~ 10% of women of reproductive age globally and can present with a variety of symptoms ranging from severe menstrual pain to fertility problems [5,6]. Affected patients can experience a significant impact on their quality of life due to chronic pain, associated depression and anxiety, and may miss school or work days [1, 3, 5].

There is typically a long delay in the diagnosis of endometriosis because the symptoms are non-specific. Until recently, histological confirmation during laparoscopic surgery has been considered the definitive method for diagnosing the disease [1]. However, recent advances in imaging techniques such as ultrasonography or magnetic resonance imaging enable diagnosis of the disease without laparoscopy [1]. In fact, the European Society of Human Reproduction and Embryology (ESHRE) describes a diagnostic process for endometriosis with and without the use of a diagnostic laparoscopy and histology [7].

The treatment of endometriosis is based on patient needs and symptoms, with most cases requiring management of endometriosis-related pain [8]. In these cases, classical treatment strategies include medical (non-hormonal and hormonal) as well as surgical ones [8]. Non-hormonal treatments, such as paracetamol or non-steroidal anti-inflammatory drugs (NSAIDs), are usually not adequate for patients requiring hormonal therapies or even opioid analgesics. Of note, hormonal therapies (e.g., progestin-only pills, GnRH analogs, combined oral contraceptives) are efficacious in reducing endometriosis-associated pain, but should be discontinued when actively attempting to conceive [8].

The surgical removal of endometriotic lesions, scar tissue and adhesions via laparoscopy is an invasive procedure with inherent risks and complications [1]. While surgery has been shown to result in the highest health-related quality of life improvements, it is not considered definitive, given the risk of recurrence [1]. As a matter of fact, 40–45% of patients who have undergone surgical removal of endometriosis experience a reoccurrence of symptoms, and 30% of patients are readmitted for surgery within 5 years of the first surgery [2].

As far as non-pharmacological treatments are concerned, the majority of patients (60%–70%) utilize some form such as acupuncture, Chinese herbal medicine, physiotherapy or nutritional strategies [9]. In addition, digital health interventions, such as apps, virtual reality goggles and wearables offer promising new tools for pain management. In fact, an increasing number of apps have been developed to provide symptom assessment and tracking, educational content and community support for patients with the disease [10,11]. Digital health interventions offer a new approach to treating chronic pain diseases [12]. In addition, apps can also record real-world data to provide insights into the effectiveness of interventions in a routine care setting. Unlike clinical trials for medical therapies, there is often no equivalent, highly regulated process for the evaluation of non-pharmacological treatments, making real-world evidence useful [12].

The ‘Endo-App’ (Endo Health GmbH, Chemnitz, Germany), a recently developed smartphone app, has already proved its efficacy in improving the physical and psychological symptoms of patients with endometriosis in randomized controlled trials (RCT) and other observational studies [13,14]. The app provides exercises and educational content for patients with endometriosis to enable multimodal pain management and reduce endometriosis symptoms [13,14]. What currently remains uncaptured is the evaluation of its effectiveness in addressing endometriosis complaints in a real-world/everyday setting. For this purpose, a retrospective study was conducted using an optional anonymous survey that was distributed to users of the app upon first subscription, after 3 months and then 6 to 7 months after the initial subscription.

## Materials and methods

### Study design

Data were collected between 19-04-2024 and 20-02-2025 using the “Endo-App”, a smartphone-based digital health tool that is fully covered by all German health insurance plans upon diagnosis of endometriosis on prescription. An optional, six-question anonymous survey was provided to users of the Endo-App on first subscription and again after ~ 3 months and ~ 6–7 months of use. Users were able to choose how many, if any, questions to answer. The answer options were either semi-qualitative/ordinal, ranked on a scale of 1–10, or in integer form, such as days on sick leave for the last 30 days.

The survey questions aimed to measure the impact of the Endo-App on endometriosis symptoms/complaints and quality of life. The following endpoints were assessed: the user’s ability to work (1 = very bad to 10 = very good), number of days missing from work over the last 30 days (0–30 days), number of days requiring medical assistance/care over the last 30 days (0–30 days), average pain levels per numeric pain rating scale (NPRS) (0 = no pain–10 = worst pain imaginable), number of days requiring pain medication over the last 30 days (0–30 days), and a self-reported ranking of the user’s general well-being on a scale of 1–10 (0 = very bad to 10 = very good).

The data sets used in this study were retrieved by independent data monitoring staff and reviewed for accuracy by another independent contractor.

### Human Ethics and consent to participate

Users of the app gave informed consent prior to participation in the survey. This included a General Data Protection Regulation (GDPR) agreement that allowed for the use of their data for anonymous research. Given the anonymous nature of the analysis, no separate ethics approval was necessary, as confirmed by the Ethics Committee of Saxony after written contact.

### Statistical analysis

In total, 143 users of the app completed at least part of the survey at 3 months and 55 users at 6 to 7 months. Survey answers were given on several numerical scales: a scale of 0–30 for questions asking for number of days out of the last 30 days; or a scale of 1–10 for questions asking users to rank their ability or pain. User responses to the survey were analyzed in MatLab.

A paired, parametric t-test was conducted for paired values where answers from the same patient were available at baseline (T0) and after 3 months (T1) or paired values of T0 and 6 to 7 months later (T2). The threshold level for statistical significance was set to 0.05, where no significant difference (ns) was defined as p > 0.05. Data were reported as mean (standard deviation [std]) with Cohen’s d as an indicator for effect size as appropriate, unless specified otherwise.

## Results

### Patient demographics

Demographic data were available for some of the patients. Due to the need to anonymize the data, it is not possible to say if the patients with demographics data answered the survey at 3 months and/ or 6 to 7 months after their first subscription. The average age [mean (std)] of patients was 34.82 (8.42) years, with an average age at first period being 12.65 (1.64) years; see Table 1. There was a large difference between the time since the first onset of pain symptoms of 12.04 (9.87) years and the time since the first diagnosis 3.77 (5.16) years; see Table 1. Regarding the relationship status of participants, 23 participants reported being married, 23 participants were single, 16 participants had a partner and were living with them and 18 participants had a partner but were not living together. Forty-six participants had regular cycles, while 25 did not have regular cycles; see Table 1. The average reported cycle length was 24.78 (8.12) days. Information on parallel interventions, such as hormonal treatment, was not retrieved as part of the survey and is hence not reported/available.

**Table 1:** Participant demographics are shown for the real-world study population that used the Endo-App

Question	Mean	Std	N, Number of patients who answered (%)
Age (years)	34.82	8.42	78
Age at first period (years)	12.65	1.64	78
Time since first diagnosis (years)	3.77	5.16	73
Time since you first experienced endometriosis pain (years)?	12.04	9.87	71
Relationship status			80
Married			23/80, 28,8%
Single			23/80, 28.8%
Partners living together			16/80, 20%
Partners not living together			18/80, 22.4%
Regular menstrual cycles			71
Yes			46/71, 64,8%
No			25/71, 35.2%
Average cycle length (days)	24.78	8.12	60

*N* number of patients

After 3 months of using the Endo-App, there were significant improvements in some symptoms.

**Fig. 1:**
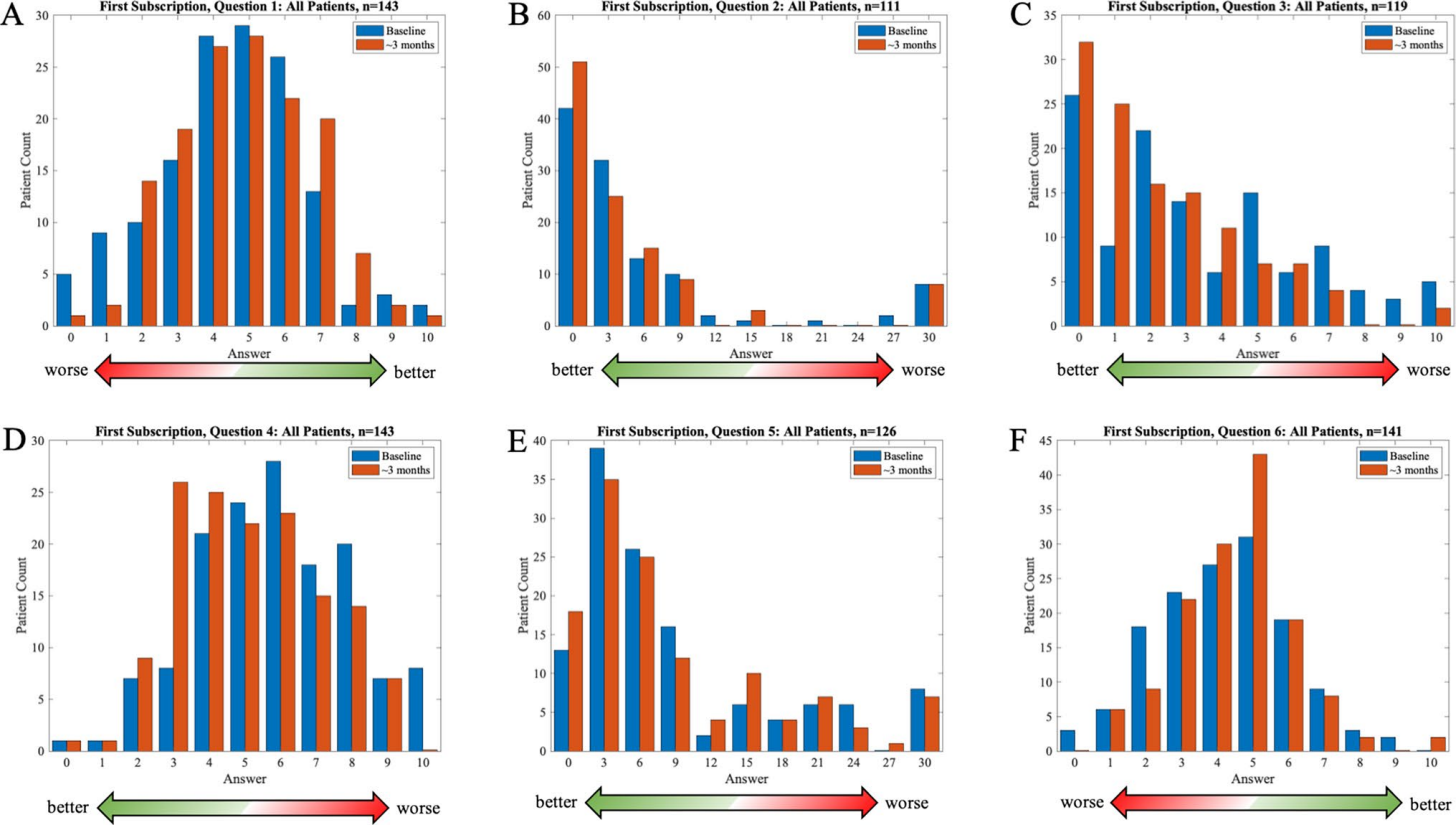
After 3 months of using the Endo-App, there was an improvement in some symptoms. **A** Rate your ability to work, *n* = 143; **B** number of sick days over the last 30 days, *n* = 111; **C** number of days over the last 30 days requiring medical assistance, *n* = 119; **D** average pain level, *n* = 143; **E** number of days requiring pain medication over the last 30 days, *n* = 126; **F** rank your general well-being, *n* = 141. T0 = baseline, T1 = survey responses 3 months after signing up to the Endo-App

Average pain levels were assessed using the NPRS scale and significantly improved at the 3-month timepoint with a pain score of 5.87 (2.12) at baseline (T0) compared to 5.07 (2.0) at 3 months (T1) (*p* < 0.0001, d = 0.28). In addition, there was a significant reduction in the number of days in need of medical assistance with a normalized score of 3.38 (2.9) at T0 versus 2.28 (2.27) days at T1 (*p* < 0.0001, d = 0.3; Fig. 1 and Table 2). While improvements were observed, there was no statistically significant difference in the scores of a user’s ability to work of 4.50 (2.07) at T0 versus 4.83 (1.88) at T1, d = 0.12, or the number of sick days users had taken with a score of 5.63 (8.47) at T0 versus 4.81 (7.86) at T1, d = 0.07. Finally, there was no significant difference in the scores for the number of days in a month where pain medication was required with 8.82 (8.48) at T0 and 8.63 (8.37) at T1, d = 0.02 and the scores for ranking general well-being with 4.21 (1.85) at T0 versus 4.48 (1.64) at T1, d = 0.11.

**Table 2:** Participant responses to survey questions after 3 months of using the Endo-App

Question	T0 mean (std)	T1 mean (std)	Pooled std	Cohen’s d	Wilcoxon T	t-test p-value	Significance
1) Rate your ability to work	4.50 (2.07)	4.83 (1.88)	2.11	0.12	0.19	0.06	ns
2) Number of sick days over the last 30 days	5.63 (8.47)	4.81 (7.86)	8.25	0.07	0.40	0.30	ns
3) Number of days over the last 30 days, requiring medical assistance	3.38 (2.90)	2.28 (2.27)	2.83	0.30	0.00	0.00	****
4) Average pain levels	5.87 (2.12)	5.07 (2.01)	2.05	0.28	0.00	0.00	****
5) Number of days requiring pain medication	8.82 (8.48)	8.63 (8.37)	7.26	0.02	0.56	0.78	ns
6) Rank your general well-being	4.21 (1.85)	4.48 (1.64)	2.03	0.11	0.13	0.12	ns

Statistical analysis: paired, parametric t-test. ns *p* ≥ 0.05, *p < 0.05, **p < 0.01, ***p < 0.001, ****p < 0.0001. T0 = baseline (answers before starting to use the Endo-App), T1 = survey responses 3 months after signing up to the Endo-App

After 6 months of using the Endo-App, further improvements were observed.

**Fig. 2:**
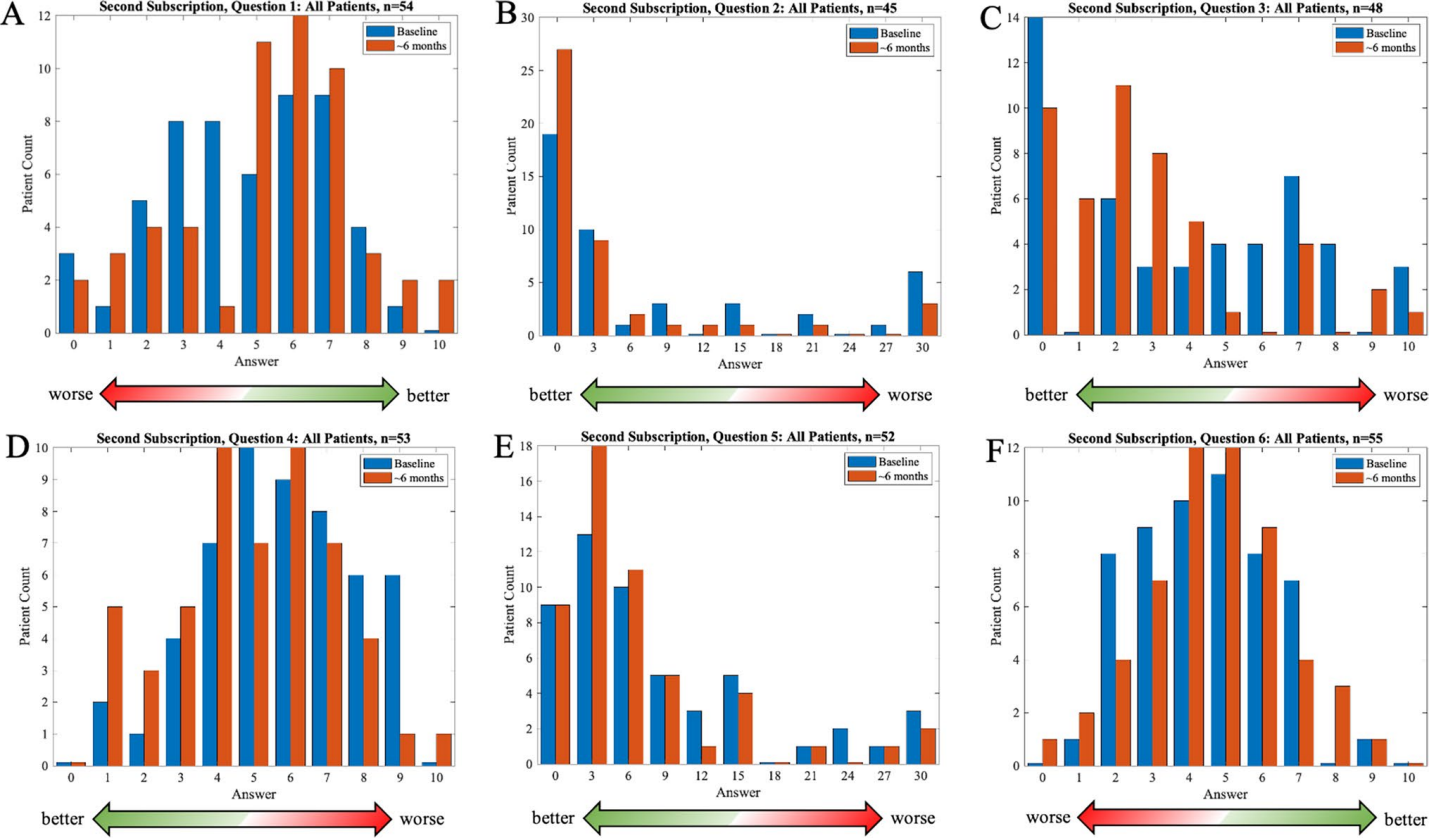
Use of the Endo-App continues to improve symptoms after ~ 6–7 months of use. **A** Rate your ability to work, *n* = 54; **B** number of sick days over the last 30 days, *n* = 45; **C** number of days over the last 30 days requiring medical assistance, *n* = 48; **D** average pain level, *n* = 53; **E** number of days requiring pain medication over the last 30 days, *n *= 52; **F** rank your general well-being, *n* = 55. T0 = baseline, T2 = survey responses 6–7 months after signing up to the Endo-App

After using the Endo-App for approximately 6 months, survey results indicated improvements in the number of sick days taken [7.82 (10.90) at T0 versus 4.22 (8.22) at T2, *p* = 0.04, d = 0.26], and the number of days requiring medical assistance [(3.92 (3.28) at T0 versus 2.77 (2.59) at T2, *p* = 0.03, d = 0.27; see Fig. 2 and Table 3)]. The scores for average pain levels at T0 were 5.77 (2.07) versus 4.92 (2.2) at T2 (*p* = 0.02, d = 0.28). There were no improvements in the reported ability to work with 4.72 (2.25) at T0 and 5.30 (2.41) at T2, d = 0.15, or the number of days pain medication was required with scores of 8.62 (8.32) at T0 and 6.65 (7.28) at T2, d = 0.18. Finally, there was no significant improvement in the ranking of general well-being, with scores of 4.45 (1.77) at T0 and 4.62 (1.89) at T2 (d = 0.06).

**Table 3:** Participant responses to survey questions after 6–7 months of using the Endo-App

Name	T0 mean (std)	T2 mean (std)	Pooled std	Cohen’s D	Wilcoxon T	t-test p-value	Significance
1) Rate your ability to work	4.72 (2.25)	5.30 (2.41)	2.50	0.15	0.11	0.10	ns
2) Number of sick days over the last 30 days	7.82 (10.90)	4.22 (8.22)	11.45	0.26	0.07	0.04	*
3) Number of days over the last 30 days, requiring medical assistance	3.92 (3.28)	2.77 (2.59)	3.47	0.27	0.04	0.03	*
4) Average pain levels	5.77 (2.07)	4.92 (2.20)	2.51	0.28	0.01	0.02	*
5) Number of days requiring pain medication	8.62 (8.32)	6.65 (7.28)	7.12	0.18	0.05	0.05	ns
6) Rank your general well-being	4.45 (1.77)	4.62 (1.89)	2.14	0.06	0.53	0.57	ns

Statistical analysis: paired, parametric t-test. ns *p* ≥ 0.05, *p < 0.05, **p < 0.01, ***p < 0.001, ****p < 0.0001. T0 = baseline (answers before starting to use the Endo-App), T2 = survey responses 6 to 7 months after signing up to the Endo-App

## Discussion

In the current study, subscribers of the Endo-App were asked to fill in an anonymous six-question survey on first subscription and again after 3 and 6 to 7 months of use. This was the first time the effectiveness of the Endo-App was tested in real-world conditions [13]. The results indicate that the use of the Endo-App might be associated with an improvement of some endometriosis symptoms, which could be sustained for 6 to 7 months of Endo-App usage. This aligns with previously published findings on the effectiveness of digital health interventions in managing chronic disease symptoms [10,11].

The availability of digital health interventions for managing chronic health conditions is increasing, and their effectiveness has been demonstrated in the management of conditions such as type 1 diabetes and endometriosis [10,11]. In Germany, the Digital Health Act of 2019 mandated the incorporation of digital health applications, such as the Endo-App, into the statutory health insurance system [15,16]. The aim was to modernize the treatment approaches available to patients. In alignment with this, the current study demonstrated significant improvements in the pain scale and in the number of days requiring medical assistance at 3 months after its subscription. Up to 6 to 7 months after the first subscription, there were significant improvements in the number of sick days in the preceding 30 days, number of days requiring medical assistance, and average pain level.

Despite the importance of real-world evidence in the assessment of digital health interventions, this study presented some limitations which should be considered. First, one limitation was the open-ended nature of the survey, which permitted participants to decide which questions to answer. Consequently, there was considerable variability in the number of responses received for each item. Notably, the greatest variation in the number of respondents was observed between the two survey time points rather than across individual questions. Some overlap may have occurred among patients who completed the survey at both time points. Because survey items were optional, the number of respondents varied across questions at each time point. The minimum number of respondents, defined as the lowest number of participants responding to any of the six survey questions, was 111 at 3 months and 45 at 6 to 7 months. The aforementioned aspects might introduce selection bias, thus, affecting the results. Patients responding to the survey might present different characteristics and/or outcomes compared to those not responding to the questions. Hence, the observed outcomes should be confirmed in future studies with larger sample sizes, duration and more strict designs.

Another limitation is that information on concurrent interventions, such as hormonal therapy, surgery, physiotherapy, or other non-pharmacological interventions, was not captured by the survey. As a result, it was not possible to account for their potential influence when evaluating the effect of the intervention, an aspect which might introduce confounding in the estimation of the effect of usage of the Endo-App per se. Finally, it should be noted that the report of this research was written as part of a non-monetary scientific collaboration between Endo Health GmbH and the Department of Gynecology, Obstetrics, and Reproductive Medicine at Saarland University Medical Center. One author (CA) is employed by Endo Health GmbH, while another (MS) serves on Endo Health’s advisory board and has received consulting fees from the company.

## Conclusions

This real-world data analysis underscores the potential positive effect of a digital application for patients with endometriosis on a variety of clinical outcomes. Future investigations should consider larger cohorts, the interaction of concomitant interventions with the app, and longer follow-up.

## Data Availability

Please contact the corresponding author for individual solutions regarding study data.

## Abbreviations

ESHRE: European Society of Human Reproduction and Embryology
GDPR: General Data Protection Regulation
NPRS: Numeric pain rating scale
NSAIDS: Non-steroidal anti-inflammatory drugs
ns: non-significant
RCT: Randomized controlled trials
Std: Standard deviation
T0: Timepoint 0 = baseline
T1: Timepoint 1 = 3 months of app usage
T2: Timepoint 2 = 6–7 months of app usage
